# Functional outcomes and quality of life after transobturatory slings: hand - made vs. commercial slings

**DOI:** 10.1590/S1677-5538.IBJU.2017.0524

**Published:** 2018

**Authors:** Danilo Budib Lourenço, Fernando Korkes, José Eduardo Vetorazzo, Silvia da Silva Carramão, Antônio Pedro Flores Auge, Luis Gustavo Morato de Toledo

**Affiliations:** 1 Departamento de Urologia, Hospital Israelita Albert Einstein, São Paulo, SP, Brasil; 2 Departamento de Urologia, Santa Casa de Misericórdia de São Paulo, São Paulo, SP, Brasil; 3 Departamento de Ginecologia, Santa Casa de Misericórdia de São Paulo, São Paulo, SP, Brasil

**Keywords:** Urinary Incontinence, Stress, Pelvic Floor, Suburethral Slings

## Abstract

Surgical correction is the most efficient treatment for stress urinary incontinence (SUI), and transobturator sling (TO) has optimal results. The high cost of commercially available sling kits makes it difficult the access in most Brazilian public health services. Hand-made polypropylene slings, on the other hand, have been previously reported. The aim of the present study was to compare the effectiveness and safety of commercial vs. hand-made polypropylene mesh slings.

Data from 57 women who underwent consecutive TO sling surgery to treat SUI were pros- pectively collected between 2012 and 2014, and divided in two groups for further compa- rison. In Group-1, 31 women underwent surgery with commercial slings. In Group-2, 26 women underwent hand-made polypropylene slings. Women were compared according to epidemiological data, perioperative evaluation, quality of life, urodynamic study, cure and complication rates. Results were objectively (stress test with Valsalva maneuver, with at least 200mL vesical repletion) and subjectively evaluated by the Patient Global Impression of Improvement(PGI-I), Visual Analog Scale (VAS) and ICIQ-SF. Success was defined as PGI-I, VAS and negative stress test.

Group-1 (n=31) and Group-2 (n=26) had a mean age of 60 vs. 58years (p=0.386). All de- mographic data were similar. The mean VLPP was 75.6cmH2O vs. 76.6cmH2O (p=0.88). The mean follow-up was 24.3 vs. 21.5months (p=0.96). Success rates were 74.2% vs. 80.2% (p=0.556), with ICIQ-SF variation of 12.6 vs.15.5 (p=0.139) and PGI-I of 71% vs. 80% (p=0.225). There was only one major complication (urethrovaginal fistula in Group-1). In conclusion, handmade and commercial slings have similar effectiveness and safety. The manufacture technique has important key-points stated in the present manuscript.

## INTRODUCTION

Urinary incontinence is a frequent complaint among women after the fourth decade of life, with an estimated prevalence of 25% in this subset of women in Brazil (1). Stress urinary incontinence (SUI) represents two-thirds of the cases (2), with similar data worldwide (3). It is definitely a major public health issue and affects primarily the quality of life of this female population (4). The association of increased intra-abdominal pressure with dysfunction of urethral occlusion generates the SUI (5). Throughout history, several techniques have been developed. Burch procedure was described in 1961 and was considered the gold standard procedure for stress urinary incontinence. In 1996, Ulmsten et al. demonstrated good results with the retropubic Tension-free Vaginal Tape (TVT). Currently, the midurethral slings represent the most used worldwide procedures for the surgical treatment of SUI (6). Delorme, in 2001, introduced the transobturator sling (TO) technique, characteristically avoiding a significant urethral tension and not violating the retropubic space (RP). It has similar success rates, with a lower number of complications (7).

There are different types and brands of commercial slings, each of them with its peculiarities and advantages. A major caveat of these commercially available slings is their elevated cost (4 4). About 70% of the Brazilian population is treated in the public health system. Most hospitals in the public health system do not have access to commercially available slings due to their relatively high prices and no reimbursement of the tape by the public health system. For these women, classical procedures are the only option, as open surgery or autologous fascial slings. Our group has previously demonstrated that autologous slings are a good alternative for mesh slings, even though a slightly higher complication rate can be expected with this technique - noteworthy bladder outlet obstruction (8). For TO slings, a good low-cost alternative that has been previously described is the hand-made polypropylene mesh slings. They seem to have similar results and safety outcomes in previous small series (9, 10). Costs, however, for handmade slings are around 1/10 of commercial counterparts. This huge difference could make accessible this valuable technique in the public health system setting in our country. Currently, hand-made slings are off-label procedures. Even though safety of polypropylene mesh in human procedures have been largely established (11), specifically for vaginal procedures there are legal caveats of a new and not yet established technique. All patients in our series were advised of this limitation and the potential risk of complications (infection, extrusion, obstruction and others) and agreed signing an informed consent form. The objective of the present study was to prospectively compare the efficacy, safety, results and quality of life for women treated for SUI with commercially available vs. handmade polypropylene mesh TO slings.

## PATIENTS AND METHODS

The present study has evaluated 57 women consecutively treated for predominant SUI with TO slings at the same institution, between January 2012 and December 2014. Exclusion criteria were previous SUI surgery and concomitant surgery for associated pelvic conditions. These women were separated in two groups, according to the type of mesh utilized in each surgery. Commercial meshes were used every time when they were available. Availability of these meshes was possible only when companies donated it for training and demonstration purposes, since it is not a standard product in the Brazilian public health system.

**Figure 1:**
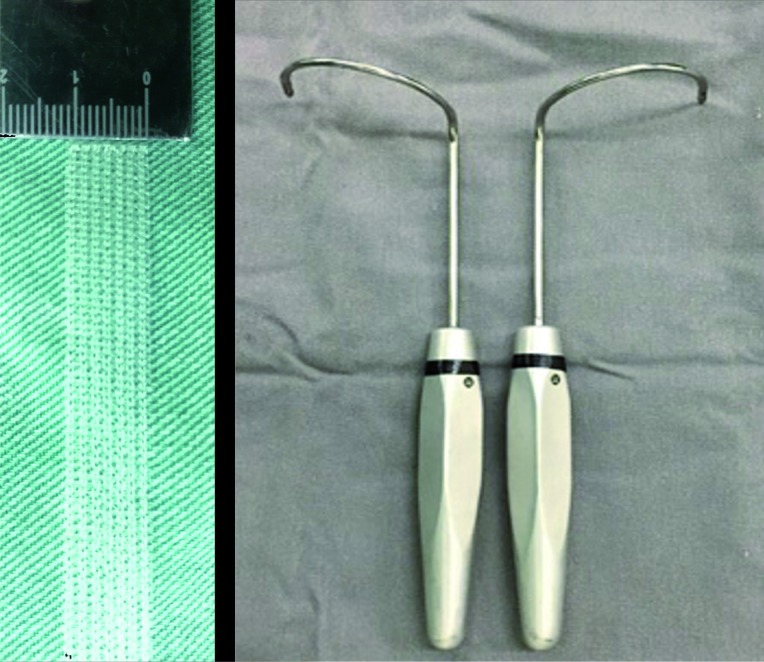
Hand-made polypropylene mesh / Needles.

Commercially available slings used were the Obtryx® (Boston Scientific, United States) and Unitape T Plus® (Promedon, Argentina). Hand-made slings were manufactured during each procedure, cutting a polypropylene mesh (Intracorp®-Venkuri, Brazil) to proper size. We have used the same macroporous and monofilament polypropylene mesh in every procedure. Only one sling was obtained from each mesh. To assure reproducibility, all meshes were cut in the lowest elasticity direction, parallel to their lines, counting a total of seven sewing lines wide, resulting in a 1.1cm x 30.5cm mesh (Figure-1). Meshes were cut in a sterile environment in the operating table, immediately prior to surgery, and the remaining mesh was not further used. A permanent transobturator needle was used to pass the sling according to the technique previously described by Delorme et al. (Figure-1) (7). Despite the difference between the meshes, the same surgical technique was performed in both groups. All patients were operated by the same surgeon (LGMT).

Every time there was a commercially sling available, the next patient waiting for surgery received it. When there was no commercial sling available, the next women waiting for surgery was offered the manufactured one as an alternative to other SUI procedures, explaining risks and benefits and treatment. After decision, procedures were conducted when a detailed informed consent form was signed.

All women underwent preoperative clinical evaluation and urodynamic study (in accordance with ICS guidelines). They were evaluated for demographical data (age, height, weight, body mass index, number of pregnancies, vaginal births, caesarean, forceps and abortion, time in years of menopause, hormone use-topical or systemic) and clinical outcomes (type of incontinence, use and number of pads, voiding and storage symptoms, urinary tract infection, dyspareunia, operative time, length of hospital stay, intra and postoperative complications, time with bladder catheter and reoperations). The International Consultation on Incontinence Questionnaire-Short Form (ICIQ-SF) was applied pre and postoperatively (12). Both the Patient Global Impression of Improvement (PGI-I) and Visual Analog Scale (VAS) for treatment satisfaction were applied postoperatively.

A questionnaire was applied pre and postoperatively every three months. The last evaluation of each patient was considered as the definitive results. All surgeries were performed under spinal anesthesia, with antibiotic prophylaxis with cefazolin for 24 hours. All procedures were performed applying the transobturator foramen technique, and manufactured slings were placed with the aid of a permanent needle (7).

Outcomes were obtained through clinical evaluation, questionnaire and stress test, and were divided into objective and subjective success. A stress test was performed with a full bladder and was considered successful when it was negative. When negative, tests were only considered valid if the patient voided at least 200mL after testing. Subjective success was considered when women considered themselves very much better or much better in the PGI-I and scored ≥8 on VAS for satisfaction.

Statistical analysis was performed by skilled professional, using SPSS 13.0v. Student's t test or Mann-Whitney test were used for continuous variables, according to the distribution, parametric/non-parametric. Pearson's Chi-square and Fisher's exact test were used for categorical variables. A significance level α equal to 5% was considered. The present study was approved by the Institutional Review board (CEP-process n° 040686/2015).

## RESULTS

Fifty-seven women were evaluated, 31 in Group-1 and 26 in Group-2. Both groups were similar according to demographic and preoperative data (Table-1 and Table-2).

**Table 1:** Demographic data (meanstandard deviation).

	Commercial Sling (n=31)	Hand-made Sling (n=26)	^p^
Age (years)	60±10	58±10	0.386
Height (cm)	159±7	160±6	0.710
Weight (Kg)	70.3±11.4	73.2±15.6	0.420
BMI	27.8±4.6	28.5±4.9	0.609
Menopause (years)	17.9±10.0	12.3±8.7	0.061
Pregnancy	4.2±2.5	3.3±1.8	0.143
Vaginal Birth	2.6±2.0	1.8±2.2	0.133
Cesarean section	0.8±0.9	1.1±1.3	0.512
Forceps	0.0	0.1±0.3	0.054
Abortion	0.8±1.2	0.3±0.5	0.079

**BMI** = Body Mass Index

**Table 2:** Preoperative urinary symptoms. Data expressed in (mean±standard deviation) or n (%).

		Commercial Sling (n=31)	Hand-made Sling (n=26)	P
Use of Pads		25 (80.6%)	21 (80.8%)	0.991
Number of pads		3.9±2.3	3.4±1.6	0.449
Genuine SUI		5 (16.1%)	10 (38.5%)	0.057
Mixed SUI		26 (83.9%)	16 (61.5%)	0.057
Recurrent UTI		11 (35.5%)	07 (26.9%)	0.489
Dyspareunia		10 (32.3%)	08 (30.8%)	0.904
**Urodynamic Study**				
	VLPP (cmH_2_O)	75.8±30.1	76.6±34.7	0.928
	VLPP<60	11 (35.5%)	9 (37.7%)	0.841
Detrusor overactivity		8 (25.8%)	4 (16.7%)	0.412

**VLPP** = Valsalva Leak Point Pressure; **SUI** = Stress Urinary incontinence; **UTI** = Urinary Tract Infection

Intra and postoperative data have been reported in Table-3 and Table-4. Operative time and complications were similar between both groups. Length of hospital stay was slightly higher for Group-1 (1.2 vs. 1.0, p=0.009), even though not clinically significant.

**Table 3:** Surgical data. Data expressed in (mean±standard deviation) or n (%).

	Commercial Sling (n=31)	Hand-made Sling (n=26)	^p^
Operative time (min)	33.4±8.4	32.9±8.0	0.731
Lenght of stay(days)	1.2±0.5	1.0±0.1	0.009*
Intraop. Compl.	0	0	-
Postop. Compl.	1 (3.2%)	0 (0%)	1.000
Time with catheter (hours)	30.6±83.5	21.0±3.9	0.563
Reoperation	1 (3.2%)	0 (0%)	1.000

**Table 4:** Postoperative clinical data. Data expressed in (mean±standard deviation) or n (%).

	Commercial Sling (n=31)	Hand-made Sling (n=26)	^p^
Follow-up (months)	24.4±15.5	21.5±10.5	0.962
Use of Pads	10 (32.3%)	4 (15.4%)	0.140
Number of pads	1.9±1.2	1.0±0.2	0.097
UTI	3 (9.7%)	4 (15.4%)	0.080
Dyspareunia	5 (16.1%)	1 (3.8%)	0.205

**UTI** = Urinary Tract Infection

Both subjective and objective success rates were similar between both groups according to all scores evaluated (Table-5). Patients with unsuccessful procedures (PGI-I less than 7, in a maximum scale of 10, or failure in the objective test) had similar demographic data, but all of them had preoperatively mixed incontinence, a well-known factor of bad prognosis. A relatively high rate of preoperative dyspareunia was observed (almost one third). De novo dyspareunia after the procedure was 6.4% in the commercial group and 3.8% in the propylene mesh group. Curiously, 4 patients in the commercial group, that even had objective and subjective success, still use a pad in postoperatory, as for insurance.

**Table 5:** Success rates. Data expressed in (meanstandard deviation) or n (%).

	Commercial Sling	Hand-made Sling	^p^
Objective Success	23 (74.2%)	22 (80.2%)	0.556
Subjective Success	22 (71%)	22 (80.2%)	0.220
ICIQ-SF pre	17.9±3.1	19.8±1.5	0.002*
ICIQ-SF post	5.2±6.1	4.1±5.1	0.645
ICIQ-SF improvement	12.6±7.4	15.6±5.1	0.139
Satisfaction (VAS)	7.7±3.0	9.0±1.1	0.160
PGI-I (scores 1 and 2)	22 (71%)	22 (80.2%)	0.225

**ICIQ-SF**=International Consultation on Incontinence Questionnaire-Short Form; **VAS**=Visual Analog Scale; **PGI-I**=Patient Global Impression of Improvement

There was only one Clavien-Dindo 3 complication in Group-1, a woman postoperatively diagnosed with urethral injury, requiring reoperation. This patient had a long hospitalization time and remained with an indwelling bladder catheter for 20 days.

## DISCUSSION

Urinary incontinence is a major public health issue. Several surgical techniques have been used historically, but mesh slings have gained worldwide acceptance since it is highly effective, technically easy and with low complication rates. The TO sling technique has high success rates whereas offering low complications rates (7, 13).

Previous studies have demonstrated in a small number of patients that commercial and manufactured polypropylene slings have similar results and complication rates after 90 days, when placed through the retropubic route. Bladder perforations occurred equally in both groups (9). TO technique has also been described with handmade slings. The low-cost alternative polypropylene mesh slings seem to have similar results and safety in previous small series (9, 10).

Our study has some important findings. First, success rates were similar between both groups, either when considering objective (ranged from 72-80%, p=0.0556) and subjective outcomes (71-80%, p=0.220).

Additionally, ICIQ-SF demonstrated similar improvement (Table-5). In a retrospective study, Cifti et al. have demonstrated a success rate ranging from 74% to 80%, similar between both groups in a twelve-months follow-up (10). Cure rates after sling surgery vary in the literature from 70 to 100% and are influenced by several factors such as technique modifications, incontinence severity, parity, surgeon's experience, diversity of patients and subjective criteria, which make it difficult to compare results (6). With polypropylene slings, a 80% efficiency rate in the first five years and 63% after 11 years of follow-up have been demonstrated (13). For TO slings, Sivasglioglu et al. have demonstrated a cure rate of 85% after five years of follow-up (14). These results are similar to what we have found in the present study.

Second, quality of life was similar between both groups. ICIQ-SF domains and satisfaction scale for patients from both groups were similar (Table-5). Even though the similar success rates could predict that satisfaction, we have accessed these results objectively.

Third, complications were similar between both groups. We had a low complication rate in our patients. No intraoperative complications occurred, and there was only one postoperative complication (urethro-vaginal fistula) during the 23 months of follow-up period. This complication occurred in one patient from the commercial sling group after three weeks of follow-up. She was re-operated and the sling was partially removed. In the Turkish study, extrusion occurred in 14.6% of cases in the hand-made group vs. 1.6% of cases in the commercial group (10). In that particular study, authors have cut 15 slings from each polypropylene mesh and re-sterilized it (10). We believe that the technique we have used to manufacture the slings are a strength of the present study. There are dozens of different meshes commercially available, with distinctive characteristics, such as elasticity, pore size, thickness and sewing shape. Thus, the results of the hand-made sling cannot be generalized. The characteristics of the mesh and the variability in sling cutting and implantation can significantly influence success rates and complications. We have used the same macroporous and monofilament polypropylene mesh in every procedure and just one sling was cut from each mesh. Meshes were cut always at the same way, with sterile precaution, in the operating table, and was immediately used. These cautions might be the explanation for the different complication rates between our study and the Turkish study. Our overall complication rate of 1.7% was similar to reported in larger midurethral slings series (15).

Our study has some limitations. First, due to its retrospective nature, a relatively small number of cases, and a short follow-up period, it cannot bring any definitive conclusion. However, since there is a paucity of studies and our study has demonstrated a different result from the previous ones, it has become very relevant. The present technique allows women from the Brazilian public health system to have access to polypropylene slings in a 10 times less expensive fashion than the commercially available ones.

The length of hospital stay was statistically longer for the commercial group (0.2 days longer), even though this might be due more of a result of bureaucratic issues, and does not pose a clinical relevance. The preoperative ICIQ-SF was higher in the hand-made group, what could suggest that the groups were different. However, this 2-point difference doesn't have a clinical relevance. Moreover, the variation between pre and post-operative ICIQ-SF was similar between groups.

In conclusion, the hand-made polypropylene sling manufactured according to our technique demonstrated to be as efficient and safe as the commercially available kits, with a low complication rate during a two-year follow-up.
